# Intravital imaging of host–parasite interactions in skin and adipose tissues

**DOI:** 10.1111/cmi.13023

**Published:** 2019-04-03

**Authors:** Mariana De Niz, Gavin R. Meehan, Nicolas M.B. Brancucci, Matthias Marti, Brice Rotureau, Luisa M. Figueiredo, Friedrich Frischknecht

**Affiliations:** ^1^ Institute of Cell Biology, Heussler Group University of Bern Bern Switzerland; ^2^ Wellcome Centre for Integrative Parasitology University of Glasgow Glasgow UK; ^3^ Malaria Gene Regulation Unit, Department of Medical Parasitology and Infection Biology Swiss Tropical and Public Health Institute Basel Switzerland; ^4^ University of Basel Basel Switzerland; ^5^ Trypanosome Transmission Group, Trypanosome Cell Biology Unit, Department of Parasites and Insect Vectors, INSERM U1201 Institut Pasteur Paris France; ^6^ Faculdade de Medicina, Instituto de Medicina Molecular João Lobo Antunes Universidade de Lisboa Lisbon Portugal; ^7^ Integrative Parasitology, Centre for Infectious Diseases University of Heidelberg Medical School Heidelberg Germany

## Abstract

Intravital microscopy allows the visualisation of how pathogens interact with host cells and tissues in living animals in real time. This method has enabled key advances in our understanding of host–parasite interactions under physiological conditions. A combination of genetics, microscopy techniques, and image analysis have recently facilitated the understanding of biological phenomena in living animals at cellular and subcellular resolution. In this review, we summarise findings achieved by intravital microscopy of the skin and adipose tissues upon infection with various parasites, and we present a view into possible future applications of this method.

## 1 INTRODUCTION

Over the course of almost a century, the implantation of imaging windows on animals for microscopic observation has been widespread, and within this time frame, it has evolved significantly. Now coined “intravital microscopy” (IVM), the technique of using optical windows to visualise phenomena at cellular or subcellular resolution has come a long way since its first use in 1824, when it was applied to visualise the rolling of leukocytes on the vascular endothelium of living frogs (Dutrochet, 1824; Wagner, 1839). These studies and an additional series of first discoveries were key for understanding endothelial physiology. Various animal models (including worms, fish, insects, amphibians, reptiles, and mammals) have been imaged since by IVM. In parallel, a series of technological breakthroughs over the past few decades in the field of physics, optics, and genetics have transformed IVM from an exotic tool to a commonly used platform to dissect processes of health and disease in living animals. Advances in image analysis have also transformed the use of IVM from mostly qualitative to yield quantitative results (reviewed by Coombes & Robey, 2010).

In parasitology, even though animal models exist for a considerable range of parasites, many questions remain understudied in vivo. In this review, we present findings that have been achieved by IVM focusing on vector‐borne parasites. Furthermore, we provide insights into the optimised methodology, surgical procedures, imaging, and anaesthetic techniques that have made these findings possible. We aim to convey the relevance of IVM to the parasitology community, given the significant findings it has already allowed. Equally importantly, it has allowed us to reassess ideas and questions proposed by in vitro studies, which were not possible to observe in vivo, or not possible to quantify. With current technology, we have been able to revisit these questions, and in many cases find unexpected features relevant to pathology, or cell biology. The use of animals and live imaging in particular has been extremely valuable for the parasitology field in the absence of relevant in vitro or in silico models that can faithfully reproduce host–parasite interactions of clinical relevance to human health.

## 2 BIOLOGICAL RELEVANCE OF THE SKIN AND ADIPOSE TISSUE FOR PARASITOLOGY

Parasites responsible for human diseases are transmitted in various ways, including through ingestion of contaminated water, food, or soil; sexual contact; transcutaneous penetration, or bites of infected vectors. Vector‐borne parasites introduced into the skin during a bite include *Plasmodium* spp. and *Wuchereria* and *Brugia* spp. (filarial worms) transmitted by mosquitoes; *Trypanosoma brucei* spp. transmitted by tsetse flies; *Onchocerca volvulus* transmitted by *Simulium* (black) flies; and *Leishmania* spp. transmitted by sandflies. Other parasites entering the host skin by routes other than vector bites include *Trypanosoma cruzi*, transmitted in triatome faeces or urine, *Schistosoma* spp. where the swimming cercariae can actively penetrate the skin, and *Dracunculus* spp. (Guinea worms), which is ingested orally, but subsequently invades subcutaneous tissues, from where it slowly egresses upon contact with water. However, the skin is much more than a route of entry into the host, as most parasites spend at least part of their existence there and often initiate a first host response. The skin can also serve as an anatomical reservoir of parasites and is a recurring theme in arthropod‐borne human diseases, probably because skin invasion for enhanced transmission to the next host is likely a powerful evolutionary force.

**Figure 1:**
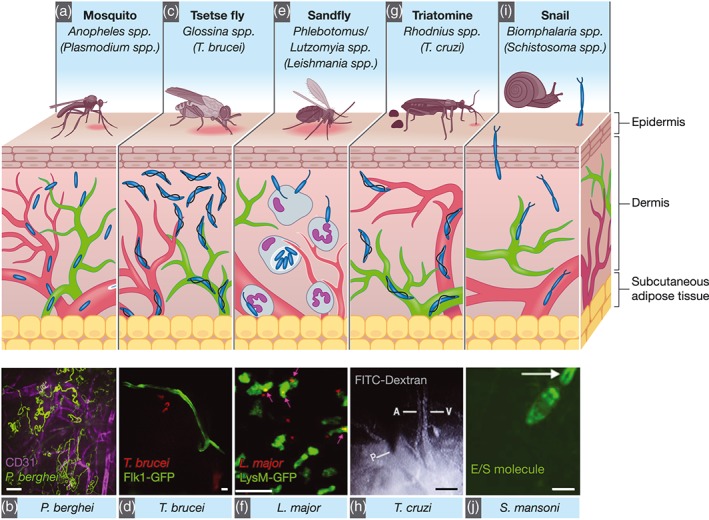
Anatomically, the skin is divided into three compartments: the epidermis (an avascular layer mostly composed of keratinocytes and Langerhans cells), the dermis (highly perfused by blood [red] and draining lymph vessels [green]). (a) Plasmodium parasites are transmitted by infected Anopheles mosquitoes. Inoculated Plasmodium sporozoites can either remain in the skin, be transported to lymph nodes via lymph, or to the liver via blood vessels. Sporozoites show different migration dynamics at different skin sites or when in proximity to blood capillaries. (b) Maximum projection of P. berghei sporozoite tracks (green) in the proximity of blood vessels (CD31, magenta). Scale bar = 25 μm (Hopp et al., 2015). (c) Trypanosoma brucei parasites are transmitted by the bite of infected tsetse flies (Glossina spp.). They first develop in the skin at the bite site before they reach the blood and lymph vasculatures, and the entire dermis is an important parasite reservoir. (d) Extravascular trypanosomes (red) imaged in the skin vessels of Flk1‐GFP mice (green) using spinning‐disc confocal microscopy. Scale bar: 10 μm (Capewell et al., 2016). (e) Leishmania spp. are transmitted by the bite of infected Phlebotomine or Lutzomyia sandflies. Neutrophils are recruited to the bite site and are crucial for the dissemination of parasites. (f) Two‐photon IVM still from a LysM‐GFP mouse (neutrophils, green) 2 hr after infection with Leishmania major (red). Scale bar = 20 μm (Peters et al., 2008). (g) Trypanosoma cruzi is transmitted via the faeces of the triatomine Rhodnius prolixus. (h) Vascular permeability as shown by FITC‐Dextran leakage from the blood vessel upon insect probing. Scale bar = 100 μm (Soares et al., 2014). (i) Schistosoma spp. undergo asexual reproduction in freshwater snails (Biomphalaria spp.). Schistosoma spp. cercariae are motile and invade the host skin. (j) Schistosoma excretory/secretory molecule release (using a fluorescent tracer [green]) during parasite skin invasion. Scale bar = 100 μm (Paveley et al., 2009)

The skin is the largest organ of the human body, and it represents the first line of immunological defence against many infections, with extensive crosstalk between epithelial, stromal, and immune cells to ensure homeostasis. Most parasites have developed mechanisms to evade detection and successfully establish an infection either in the skin itself or elsewhere in the host. Anatomically, the skin can be divided into three distinct compartments: the epidermis, which is an avascular layer mostly composed of keratinocytes and Langerhans cells; the dermis, which is highly perfused by blood and draining lymphatic vessels (Figure 1); and the subcutaneous adipose tissue. The structure of the skin provides an interface between the vascular and lymphatic circulations, as well as the interstitial space. The latter is a fluid‐filled anatomical compartment defined by a complex lattice of collagen bundles, found within and between tissues including the dermis (Benias et al., 2018). Until recently, the physiological importance and extent of the interstitium had been largely understudied, yet this compartment is very likely to be of relevance for host–pathogen interactions defining phenomena such as extravasation and sequestration of different parasites.

Direct in vivo and in situ imaging of the skin has facilitated the understanding and identification of host and pathogen factors relevant for effective parasite transmission upon vector biting (or parasite traversal itself, as is the case of, e.g., *Schistosoma* spp.). They have also allowed the study of general aspects of parasite development including proliferation, migration, and interactions with the host immune system.

A tissue that together with the skin has gradually gained momentum and interest for its relevance as a parasite reservoir is the adipose tissue (reviewed by Tanowitz, Scherer, Mota, & Figueiredo, 2017), although its exact function(s) have not yet been established. In various fields of research, the adipose tissue went from being regarded as an inert site of energy storage or “fat,” to being considered now as a complex, multicomponent site of paramount relevance to metabolism and systemic immunity (reviewed by Tilg & Moschen, 2006). This importance has permeated into parasitology, and it is possible that the adipose tissue modulates/impacts on parasite biology. It might serve as a source of nutrients, allow modulation of immunity and pathology, and/or support transmission (in the case of subcutaneous adipose tissue). Coincident with the change in conception of the relevance of the adipose tissues, observations of some parasites at this anatomical site were already reported decades ago but few functional, dynamic, or IVM studies were pursued, or reported until recently. The potential of IVM to this tissue is further discussed in this review.

## 3 OPTICAL WINDOWS AND DIRECT IMAGING: IVM OF THE SKIN AND ADIPOSE TISSUE

**Figure 2:**
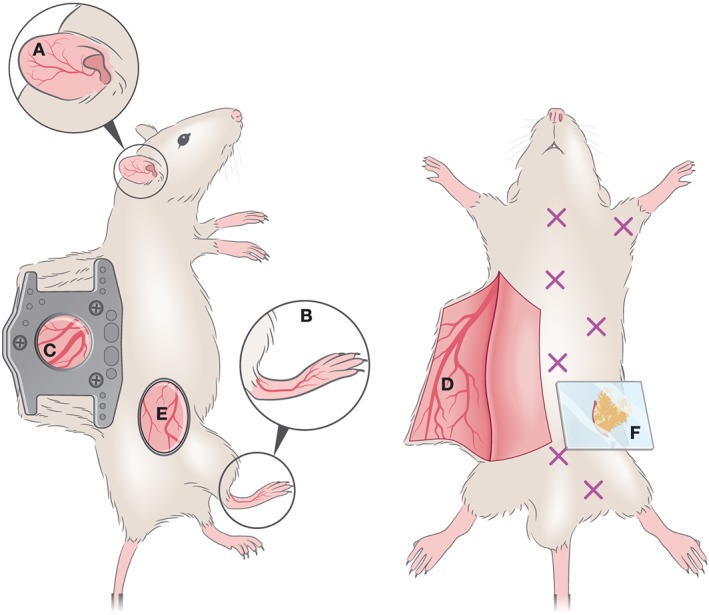
Optical windows and imaging chambers for skin and adipose tissue visualisation by IVM. Non‐invasive methods include (a) ear pinna imaging and (b) foot‐pad imaging. A semi‐invasive method (c) includes the dorsal skinfold chamber, which requires the surgical implantation of two titanium or polymer frames that can hold a ring with a glass coverslip through which imaging is performed. An invasive method is the generation of a skin flap (d), whereby a skin flap is generated, exposing a large imaging area. This procedure is invasive and terminal. A less commonly used method for IVM imaging is the skin flank (e) which requires the generation of an incision at a dorsolateral location, and either direct imaging or mounting on a stainless steel disc for stable image acquisition. For adipose tissue imaging, various types of window exist to visualise various depots (marked by X). To image the perigonadal adipose tissue, a terminal lower abdominal window (f) was generated

IVM of the mouse skin includes non‐invasive methods such as imaging of the ear pinna, footpad (Figure 2a,b), or tail; semi‐invasive methods such as the dorsal skinfold chamber (Figure 2c) or invasive methods such as the skin flap (Figure 2d). Additionally, a relatively less explored location that has been successfully imaged by IVM is the skin flank (Figure 2e). The advantages and limitations of all methods are summarised in Table 1. Skin at distinct anatomical sites exhibits important differences in, for example, thickness, composition, vascularization, and cytokine profiles. In recent years, several groups have investigated the relevance of cellular heterogeneity across skin sites with tools including IVM (Driskell et al., 2013; Jain & Weninger, 2013; Tong et al., 2015). This led the generation of a three‐dimensional immune cell atlas of mouse skin (Tong et al., 2015), demonstrating different densities of adaptive and immune cell populations at various depths of the epidermis and dermis; functional cellular niches in specific anatomical locations differing in the tail, ear, flank, and feet; and the relevance of structures such as the vasculature, and fibroblast‐rich networks of collagen and elastin, for variations in resident immune cell populations. Equally, cutaneous immune responses mediated by Langerhans cells (which act as potent antigen presenting cells) have been shown to vary dramatically across skin sites, impacting T cell responses, and immunisation outcomes (Wang et al., 2008). Importantly, these and other findings of skin cell heterogeneity have helped reconcile disparate results across studies in other fields, which used different skin locations for observations. Although few studies in parasitology have directly considered these differences, they are very relevant in the context of infection and should therefore influence the choice for imaging by IVM.

**Table 1:** Summary of windows for skin and AT imaging

Technique	Key points	Complexity	Advantages	Limitations
Footpad	Intact skin. Hair removal necessary to avoid autofluorescence.	Low	• Simple to perform. • Not invasive. • Longitudinal imaging through various days. • Saves animals (consistent with 3Rs).	• Autofluorescence if hair present. • Limited vascularization and imaging area.
Ear pinna	Intact skin. Hair removal necessary to avoid autofluorescence.	Low	• Simple to perform. • Not invasive. • Longitudinal imaging through various days. • Saves animals (consistent with 3Rs).	• Autofluorescence if hair present. • Limited vascularization and imaging area. • Rich in cartilage but poor in subcutaneous adipose tissue.
Tail	Intact skin. Hair removal necessary to avoid autofluorescence.	Low	• Simple to perform. • Not invasive. • Longitudinal imaging through various days. • Saves animals (consistent with 3Rs).	• Autofluorescence if hair present. • Poor in subcutaneous adipose tissue.
Dorsal skinfold chamber	Semi‐invasive. Requires the surgical implantation of a chamber, exposing a small skin area. The chamber remains in place and allows longitudinal imaging with recovery of the mouse.	High Important to cause as little damage as possible: the animal should survive surgery, implantation and multiple rounds of imaging.	• Longitudinal imaging through various days. • Reduces use of animals (3Rs). • Different vascular environment as that presented in more accessible skin sites.	• Could present postsurgical complications including infection and inflammation. • Chronic chamber may introduce artefacts. • Care should be taken to prevent pain.
Skin flap	Exposure of a large skin area by generating a skin flap that can be imaged through a simple window (e.g. a coverslip). It is invasive and terminal.	Medium to high Important to cause as little damage as possible to avoid bleeding or artefacts during imaging	• Large imaging area. • Different vascular environment as that presented in more accessible skin sites.	• It is terminal. Possibility of ischaemia.
Skin flank	Relatively easy access. Exposure of a relatively large skin area.	Medium to high Important to cause as little damage as possible to avoid bleeding or artefacts during imaging	• Large imaging area • Different vascular, nerve, and lymphatic environments as those presented in more accessible skin sites.	• In some modalities, it is terminal.
Abdominal window for AT access	Exposure of a large AT area that can be imaged through a simple window	Medium to high Important to cause as little damage as possible to avoid bleeding or artefacts during imaging	• Large imaging area. • Away from heart (motion artefacts avoided).	• It is terminal.

Each of the imaging sites for skin IVM mentioned above has specific advantages and limitations. Ear and footpad imaging (Figure 2a,b) allow observation over long periods of time. It is relatively easy to perform due to the non‐invasive nature of the procedure, and that neither site is heavily affected by cardiorespiratory motion. In both cases, careful hair removal is necessary to minimise autofluorescence and to prevent absorption of laser light by hair. Both sites allow non‐invasive, easily accessible longitudinal imaging of complex processes including stem cell dynamics via imaging hair follicle development, and the contributions of stem cell populations to tissue regeneration (Pineda et al., 2015). Protocols developed for longitudinal imaging in the ear pinna are a strong alternative to more complex windows relevant for other skin locations, such as dorsal or ventral skin in the midbody.

Conversely, a skin flap requires surgically generating a flap to expose the inner and outer surfaces of the dorsal or ventral skin. This procedure is more invasive than ear or footpad imaging, but it provides a larger imaging surface and a different anatomical location, which might be relevant depending on the biological question addressed. If performed without the purpose of implementing an optical chamber, this procedure allows imaging once, for relatively long periods of time, but cannot extend over the course of days (Figure 2d). The more sophisticated surgical procedure for skin imaging is the dorsal skinfold chamber (Figure 2c), which has evolved significantly since its first use in 1924 (Algire, 1946; Amon, Menger, & Vollmar, 2003; Arfors, Jonsson, & McKenzie, 1970; Knappe et al., 2005; Lehr, Leunig, Menger, Nolte, & Messmer, 1993; Sandison, 1924). Modern optical windows are made of titanium (Menger, Laschke, & Vollmar, 2002). Alternatively, they can be made of non‐metallic polymer materials that allow their use with radiation and magnetic resonance imaging (Erten et al., 2010; Gaustad, Brurberg, Simonsen, Mollatt, & Rofstad, 2008) and are lighter in weight thus causing less discomfort to the mice. The method for generating an optical window for skin IVM consists of placing two symmetrical frames to “sandwich” an extended double layer of skin. The skin is covered with a removable glass coverslip, which can be incorporated into one of the frames, allowing visualisation with an objective. This set‐up allows for repetitive analysis over a period of 2–4 weeks (Lehr et al., 1993), and it requires a 2–3‐day recovery period following implantation. Finally, skin flank IVM is among the least explored methods in the context of host–pathogen interactions, yet one worth considering in future applications in parasitology given the neural, lymphatic, vascular, and cellular composition at this site. At a dorsolateral anatomical location (Figure 2e), the flank has been a preferred site in the context of immunology studies, engraftment, and viral dynamics, including that of herpes simplex virus 1 (Ariotti et al., 2012). The surgical procedure is described by (Moalli et al., 2018) and involves creating incisions at the skin flank to separate it from the peritoneum, and generating an imaging platform by inserting a stainless steel disc under the dermis.

Different types of windows can be used to perform IVM in adipose tissue depots, depending on the tissue being accessed (Figure 2f). However, only one window type was so far used in parasitology (*Plasmodium*; De Niz et al., 2016). This work used a terminal abdominal window, whereby the surgery consisted on a small incision in the dorsolumbar area and the careful externalisation of the perigonadal adipose tissue. This was followed by the careful attachment of the surrounding skin, with a removable glass coverslip that can be directly placed on the microscope stage. Alternative to the terminal window used in this study (allowing imaging for up to 8 hours), an analogue of the dorsal skinfold chamber described above exists for the abdominal wall, which can be applied to most abdominal organs including adipose tissue depots. Although surgically much more challenging, the abdominal chamber provides the advantage of long‐term, repetitive imaging in the range of 2–4 weeks.

## 4 NEW PARASITE BIOLOGY REVEALED BY IVM

### 4.1 Plasmodium spp.

*Plasmodium* development in the mammalian host, eventually causative of malaria, is initiated when a female *Anopheles* mosquito injects motile *Plasmodium* sporozoites into the skin of a host upon probing for a blood meal (Amino et al., 2006; Matsuoka, Yoshida, Hirai, & Ishii, 2002; Medica & Sinnis, 2005; Sidjanski & Vanderberg, 1997; Vanderberg & Frevert, 2004; Yamauchi, Coppi, Snounou, & Sinnis, 2007). Successful continuation of the parasite's life cycle depends on the sporozoites leaving the skin, and travelling to the liver where an enormous expansion of the parasite mass will occur via asexual replication. The idea that mosquitoes inject *Plasmodium* sporozoites into the dermis of the host, rather than directly into blood circulation, was first proposed in the 1930s. However, the in vivo investigation of the migration of sporozoites after transmission and the discovery of the dermal phase of infection only became possible in the past decade.

Excision of the bite site showed that mosquito‐injected *Plasmodium* sporozoites remained in the skin for at least 5 to 15 min, before entering the blood stream. It was hypothesised at the time that sporozoite migration to the blood vessels following inoculation was delayed either because of anti‐sporozoite antibodies or a cutaneous hypersensitivity reaction to the mosquito bite (Sidjanski & Vanderberg, 1997). While decades earlier, it was already demonstrated that anti‐sporozoite antibodies could immobilise sporozoites in vitro (Stewart, Nawrot, Schulman, & Vanderberg, 1986; Vanderberg, 1974), revisiting this question with IVM on mice immunised with irradiated sporozoites or passively immunised by injecting anti‐CSP antibodies, and subsequently infected with WT parasites revealed a striking cessation in motility within the skin (Flores‐Garcia et al., 2018). IVM showed in vivo (a) probing of the skin by the mosquito proboscis; (b) mosquito ingestion of blood either by direct puncture of a blood vessel or pool feeding from hematomas formed upon vessel rupture by the mosquito proboscis; (c) the various types of sporozoite deposits during a mosquito feed, which could be linear or fountain‐like; (d) demonstration of sporozoite migration following a brief delay after their deposition in the dermis; and (e) entry into both blood and lymph vessels (Amino et al., 2006; Vanderberg & Frevert, 2004).

Although the first IVM studies were performed in the ear pinna, various groups later imaged mosquito bites in both the ear pinna and the ventral and dorsal midline skin (Jin, Kebaier, & Vanderberg, 2007; Kebaier, Voza, & Vanderberg, 2009; Yamauchi et al., 2007). Interestingly, this consistently resulted in fewer inoculated sporozoites in the ear, but the kinetics of migration were conserved between sites. IVM also revealed that up to 15–20% of inoculated sporozoites are transported via lymphatic vessels to the lymph nodes (Amino et al., 2006; Yamauchi et al., 2007), and that a small fraction of parasites that remain in the skin can begin developing within this organ (Coppi et al., 2011; Gueirard et al., 2010). The remaining majority of sporozoites are either eliminated or successfully travel to the liver endothelium via blood vessels.

Finally, IVM has allowed studying and quantifying the speeds of sporozoite migration at different bite sites of the dermis, the dynamics of sporozoite contact with host blood vessels, and the subsequent migration of sporozoites from the skin into the bloodstream (Amino et al., 2006; Hellmann et al., 2011; Hopp et al., 2015; Figure 1a,b). These studies revealed that the sporozoite migration path is determined by the environment (Hellmann et al., 2011); that sporozoites migrate through cells using a secreted perforin‐like protein (Amino et al., 2008), and slow down when migrating in close proximity to blood capillaries (Hopp et al., 2015).

These results also informed in vitro studies at the intersection of physics, and biology (Hellmann et al., 2011; Muthinja et al., 2017), that use patterned environments to address questions that could not be answered by IVM. For example, the different migration path in the skin of ear or tail could be assigned to the different architecture of the environment (reviewed in Muthinja et al., 2018). Small differences in sporozoite migration in the skin were observed by IVM in mice lacking a specific integrin, possibly suggesting that this molecule interacts with a sporozoite surface protein (Dundas et al., 2018). Finally, a recent elegant study revealed that the sporozoite surface protein CSP protects the parasites from the action of its own perforins, which are necessary for migration through cells in the skin (Aliprandini et al., 2018).

One of the first in vivo clues of the relevance of adipose tissue to *Plasmodium* asexual blood stages came from a study using bioluminescence to characterise *Plasmodium berghei* sequestration in rodents (Franke‐Fayard et al., 2005). This phenomenon is the ligand‐mediated arrest of *Plasmodium* schizonts and trophozoites in the host vasculature of humans and is believed to be associated with many malaria complications. The human *Plasmodium falciparum* parasite sequesters by interacting with a variety of human receptors, such as CD36, ICAM, EPCR, and CSA. A study using human skin transplants on SCID mice identified the rolling behaviour of *P*. *falciparum*‐infected RBCs in living mice (Ho, Hickey, Murray, Andonegui, & Kubes, 2000). Although the *P*. *berghei* ligand for sequestration remains unidentified, recent IVM‐based work showed that the overall machinery for parasite sequestration is conserved between human and rodent *Plasmodium* spp. (De Niz et al., 2016). This work showed that *P*. *berghei* parasites lacking two central proteins for the *Plasmodium* export machinery (MAHRP1a and SBP1), lose sequestration in the adipose tissue and lungs, decreasing virulence and improving survival. IVM showed significantly different parasite numbers and dynamics at this site when comparing WT and mutant parasite lines. Specifically, nonsequestering parasite lines were found circulating within erythrocytes in the bloodstream, whereas sequestering lines were static in the vessel walls adjacent to activated macrophages. Importantly, both, sequestering and nonsequestering lines showed a strictly intravascular localisation early in infection, consistent with sequestration rather than extravasation.

### 4.2 Trypanosoma brucei

Human African trypanosomiasis (HAT), or sleeping sickness, is caused by two main subspecies of *T*. *brucei* that are exclusively transmitted through the bite of *Glossina* tsetse flies. Within the fly, *T*. *brucei* undergoes a complex developmental cycle culminating in the production of salivary metacyclic forms that can infect mammalian hosts (Rotureau & Van Den Abbeele, 2013). Like *Plasmodium* and other vector‐borne pathogens, the presence of *T*. *brucei* changes the composition of the saliva and modifies the feeding behaviour of the tsetse fly in a way that enhances the chances of parasite transmission to the host (van den Abbeele, Caljon, de Ridder, de Baetselier, & Coosemans, 2010). Tsetse flies are pool feeders and lacerate the skin of their host rather than inserting a proboscis directly into the vasculature. After causing significant local damage and inflammation at the bite site that often result in a transient chancre, the insects feed on the resultant pool of capillary blood and lymph (Bouchet & Lavaud, 1999; Goodwin, 1970). However, our knowledge on the early interface between the parasites and the host skin in vivo remained limited until recently.

Various studies have documented skin reactions to the parasite in different animal models by histology, fluorescence‐based imaging, electron microscopy (Akol & Murray, 1982; Dwinger, Rudin, Moloo, & Murray, 1988; Goodwin, 1971, 1970; Ikede & Losos, 1972; Mwangi, Hopkins, & Luckins, 1995; Mwangi, Hopkins, & Luckins, 1990; Sbarbati et al., 2010; Thuita et al., 2008), thermographic imaging, or molecular and flow cytometric methods (Caljon et al., 2016). Nevertheless, IVM recently brought new perspectives in our understanding of the crucial role of the skin in trypanosome biology. By multiple imaging approaches, including intravital imaging in the ear pinna, African trypanosomes were confirmed to first develop in the skin at the bite site before they reach the blood and lymph vasculatures. Also, the resulting neutrophil recruitment was further proven to enhance early onset of infection (Figure 1c,d). At the same time, a multiplex kinetic intravital imaging approach (Calvo‐Alvarez, Cren‐Travaillé, Crouzols, & Rotureau, 2018) revealed that the entire dermis is an important anatomic reservoir of trypanosomes where a significant number of parasites were constantly available for transmission to tsetse vectors (Capewell et al., 2016). This imaging approach consisted of combining whole animal bioluminescence imaging, spinning‐disc confocal microscopy on the ear pinna, and two‐photon microscopy on the abdominal flank, in the same animals. In parallel, a functionally adapted population of parasites was shown to occupy adipose tissues in a mouse infection (Trindade et al., 2016), and it is possible that the adipocyte‐rich hypodermal layer of the skin may attract, host, and/or maintain at least part of the skin‐dwelling trypanosome population by providing them an immunological niche and/or a stable lipid‐rich nutritive environment (Tanowitz et al., 2017).

Although the existence of a significant extravascular population of *T*. *brucei* parasite is not novel (Goodwin, 1970), their enrichment in the skin of HAT patients and asymptomatic latent carriers (Camara et al., unpublished; Capewell et al., 2016) questions our understanding of trypanosome transmission and HAT epidemiology. This is especially important when considering the possibility of a significant silent reservoir of trypanosomes in the skin of latent carriers readily accessible to tsetse vectors (Büscher et al., 2018). The presence of parasites in extravascular sites (skin, adipose tissue, and brain) for extended periods raises several fundamental questions on parasite biology. Indeed, parasites in the blood and adipose tissue appear functionally specialised as they can catabolise fatty acids only in the fat (Trindade et al., 2016). This in turn raises several questions regarding how parasites proliferate, how differentiation is triggered preferentially in transmission‐compatible tissues, how parasites migrate, and whether and how they sequester.

### 4.3 Leishmania spp.

*Leishmania* parasites are transmitted by the bite of infected *Phlebotomus* and *Lutzomyia* sandflies. The sandfly bite leads to wounding of the microvasculature, creating a haemorrhagic pool from which the sandfly feeds (Belkaid et al., 2000; Belkaid et al., 2000; Kamhawi, Belkaid, Modi, Rowton, & Sacks, 2000; Teixeira et al., 2005; Figure 1e,f). Upon infection, three types of leishmaniasis can occur depending on the infectious species: cutaneous, mucocutaneous, or visceral. Cutaneous leishmaniasis causes nodules or ulcers in the skin, often resulting in deforming scars. Mucocutaneous leishmaniasis may develop from cutaneous leishmaniasis, forming sores in the mouth, nose, or larynx. Visceral leishmaniasis affects organs including the liver, spleen, and bone marrow, and if severe, can be fatal.

Regarding visualisation of *Leishmania* transmission in the skin, IVM has been a key tool to elucidate mechanisms of entry and invasion, which were groundbreaking in the field (Ng et al., 2008; Peters et al., 2008; reviewed by Beattie & Kaye, 2011; Ritter, Frischknecht, & van Zandbergen, 2009). Although it was known that the definitive host of *Leishmania* parasites are macrophages, neutrophils were shown to be the first transient host cells in the skin (Peters et al., 2008). A combination of transgenic parasites (*L.m*.‐RFP) and transgenic mice (LysM‐GFP, a reporter for monocytes, macrophages, and neutrophils), together with two‐photon IVM of the ear pinna, showed that neutrophils slowly infiltrate into the sandfly bite site, and continue to be recruited for an extended period of time. IVM showed that *Leishmania* parasites within neutrophils were viable, and that following apoptosis of the neutrophils, parasites were released within the vicinity of surrounding macrophages (the definitive host cell), thereby contributing to the progression of the disease (Peters et al., 2008; Figure 1e,f). Importantly, upon neutrophil depletion, a reduction in viable parasites was detected, corroborating a previously suggested role for neutrophils in *Leishmania* pathogenesis (van Zandbergen et al., 2004).

Another IVM‐based study demonstrated the unique role of dermal dendritic cells in *Leishmania* sensing (Ng et al., 2008). Two‐photon IVM of the ear pinna revealed that upon *Leishmania* infection, the highly motile dermal dendritic cells ceased their motility. This was followed by (a) the extension of long and highly dynamic pseudopods or dendrites capable of tracking and engulfing parasites, (b) retraction of the pseudopods towards the cell body, and (c) incorporation of parasites into cytosolic vacuoles. This rapid cessation of motility and rapid tracking and engulfment was specific for parasites and not observed towards inert particles. Both IVM‐based studies shed light into the role of innate immune cells at the skin interface following a sandfly bite, providing important advances in the understanding of this disease, which are impossible to detect in vitro. Lastly, a more recent study used two‐photon IVM to image for the first time, the entire time course of *Leishmania major* infection in the ear skin, following the bite of an infected sand fly. This study carefully describes various events relevant to host–pathogen interactions, pathology, and parasite dynamics previously unknown. This method takes advantage of various features of IVM to perform this long‐term imaging in a non‐invasive manner, including deep penetration, and little photodamage (Carneiro, Hohman, Egen, & Peters, 2017).

The parasites so far discussed are transmitted by the direct bite of infected vectors. Additional parasites also transmitted by vector bites include the filarial worms *O*. *volvulus*—causative agent of onchocerciasis, *Wuchereria bancrofti* and *Brugia malayi*—causative of filariasis, and the *Thelieria parva* and *Babesia* parasites—causative of theileriosis and babesiosis. However, to date, no studies have used IVM to investigate these parasites. Other parasites transmitted via the skin, but not via infected bites, include *T*. *cruzi* and *Schistosoma*. IVM‐based findings on both parasites are discussed below.

### 4.4 Trypanosoma cruzi

Triatomines are the haematophagous insect vectors of *T*. *cruzi*—the causative agent of Chagas disease. Triatomines are vessel feeders, and after piercing, the host skin will probe via rapid whip‐like intradermal movements of the maxillae until a vessel is found. Like mosquitoes and tsetse flies, triatomines possess various molecules in the saliva, which reduce haemostasis and have anti‐inflammatory properties (Ribeiro, 1995; Pereira et al., 1996; Ribeiro, Schneider, & Guimaraes, 1995 (1); Ribeiro & Francischetti, 2003). Like for mosquitoes and tsetse flies, host‐to‐vector transmission occurs when the triatomine feeds on an infected individual. However, unlike other pathogens, *T*. *cruzi* is unique in that a person only becomes infected when the triatomine defecates on the host skin during or after a feed (Figure 1g). The parasites are in the faeces and enter the body through mucous membranes (i.e., eyes or mouth) or itch‐induced scratching that leads to breaks in the skin. Despite the importance of the skin as an entry site of the parasite, *T*. *cruzi* interactions at the skin interface in vivo remain poorly understood. Only two studies have used IVM in the context of *T*. *cruzi*, and both focused on the patterns of triatomine feeding and salivation at the mouse skin, rather than vector‐to‐host parasite transmission (Soares et al., 2006; Soares, Araújo, Carvalho‐Tavares, Gontijo, & Pereira, 2014). The first study used the ear pinna of mice, together with triatomine saliva labelled with acridine orange. Using epifluorescence microscopy, it was found that salivation occurs throughout all feeding phases (probing and engorgement) of the triatomine *Rhodnius prolixus* and measured the frequency of saliva emissions (Soares et al., 2006).

The second study investigated the interface between the feeding process of the triatome and the host's response at the vascular endothelium (Figure 1h). This work used epifluorescence microscopy, as well as stereomicroscopy and an electromyogram. The main findings included (a) the induction of vascular permeability alterations following a triatomine bite via injection of dyes, (b) the immediate platelet and leukocyte aggregation at the venular endothelium following a bite, and (c) the integration of imaging and analysis methods in vector and host, which enabled monitoring vessel wall pulsations to register movements during blood pumping, as well as the evaluation of blood flow through the triatomine's head (Soares et al., 2014). Although there are no IVM studies yet that investigate parasite transmission at the skin, one ex vivo study addressed the kinetics of skin penetration of *T*. *cruzi* trypomastigotes in mice (Schuster & Schaub, 2000). A drop of vector faeces or urine containing trypomastigotes was placed on the bite site of a triatomine, and the skin was surgically removed after different periods of time. This showed that the minimal exposure period necessary for infection was as little as 5 min with longer periods of exposure time correlating with higher infection rates. This showed that *T*. *cruzi* can rapidly invade the host, and that some parasites can be carried away from the bite site immediately (Schuster & Schaub, 2000) and sets the stage for similar studies as those conducted in *T*. *brucei* and *Plasmodium* to reveal the dynamics of infection.

### 4.5 Schistosoma

Various *Schistosoma* species are the causative agents of schistosomiasis. The sexual reproduction of the parasite occurs in humans as well as other hosts, whereas asexual reproduction occurs in freshwater snails (*Biomphalaria* spp.). *Schistosoma* are motile in all life stages, and this motility is relevant to their capacity to search for, and invade the host skin, and to circulate within the host following invasion. Schistosomes are phototrophic, which leads the cercariae to preferentially localise to the surface of shallow waters, where they can maximise their contact with humans (Stirewalt & Dorsey, 1974). Cercariae then respond to thermal gradients in order to find the skin of the host and then to chemical cues that allow them to complete invasion (Fishelson et al., 1992; Gordon & Griffiths, 1951; Haas, Diekhoff, Koch, Schmalfuss, & Loy, 1997; Lewert & Lee, 1954; Salter, Lim, Hansell, Hsieh, & McKerrow, 2000). Most of the interactions of *Schistosoma* with the skin have been explored using a range of imaging, molecular, and chemical methods (reviewed by McKerrow & Salter, 2002). One study used IVM to monitor *Schistosoma* interactions with the skin to understand excretory/secretory (ES) molecule release by the parasite during skin invasion (Paveley, Aynsley, Cook, Turner, & Mountford, 2009; Figure 1i,j**)**. Using carboxyfluorescin diacetate succinimidyl ester (CFDA‐SE), a fluorescent amine reactive tracer, live cercariae were labelled to investigate their interactions with innate immune cells in the mouse ear pinna. Time‐lapse confocal imaging showed that as cercariae move through the skin, CFDA‐SE‐labelled material is released via the oral sucker. This material is thought to be a mixture of digestive proteases that aid in the migration of the parasite. Ex vivo work investigating the dynamics of ES molecule uptake by macrophages and dendritic cells showed that, depending on the relative abundance of each cell type, and differential rates of antigen processing by these cells, this might be key to the success of adaptive immune priming in response to *Schistosoma* infection (Paveley et al., 2009).

## 5 FUTURE DIRECTIONS

Considering its ease, IVM of parasites in the skin has focused mainly on the use of the ear pinnae of mice. Although this has advantages in terms of accessibility and non‐invasiveness, the few studies in *Plasmodium* and *T*. *brucei* using other skin sites to image parasite dynamics have shown differences between locations. This is particularly important as the skin is physiologically different between anatomical sites and parasites may adapt to the respective environment. The use of skinfold chambers, which allow visualisation within a much larger surface area, for a much longer period of time will be a useful tool to study new tissue locations. Interestingly, most IVM studies have focused on vector‐to‐host transmission, with only one having imaged host‐to‐vector transmission after a bite. The importance of the skin and adipose tissue as reservoirs and for disease pathology remains largely obscure and requires more attention in the future.

Technological advances including the increasing range of available fluorescent reporter mice, and transgenic parasites expressing different fluorescent proteins alongside tagged genes potentially involved in transmission will lead to further advances in our understanding of parasite biology. These technologies, in combination with the use of single‐cell transcriptome analysis and immune profiling, may reveal new tissue‐specific interfaces between parasites and their hosts that have not been observed previously.

An exciting area of IVM that is becoming more popular for parasite research is the use of humanised models. Although conventional animal models are invaluable in providing insights into parasite behaviour, they cannot fully replicate human disease due to differences in parasite strains, cell morphology, and humoral immune responses. As a result, there has been a drive in recent years to examine human parasites directly. One way to achieve this is through the use of humanised models. By incorporating human tissues and stem cells into immunocompromised animals, these models successfully mimic human physiology allowing human parasites to be studied in an in vivo environment. They have been particularly popular for studying *P*. *falciparum* infection as they allow certain aspects of malaria to be studied in a way not possible using murine *Plasmodium* spp. (reviewed by Minkah, Schafer, & Kappe, 2018).

One of these aspects is the sequestration of the *P*. *falciparum* asexual stages in the microvasculature. Although this also occurs in certain murine malaria infections, the binding ligand of *P*. *falciparum* is unique, limiting our abilities to study this behaviour closely. This has been addressed in a number of studies by utilising mice implanted with human skin grafts. Through IVM, they have observed the rolling and adherence behaviour of parasitized red blood cells in both postcapillary venules and arterioles and demonstrated that this behaviour could be reversed using antibodies that blocked the parasites binding ligands (Ho et al., 2000; Yipp et al., 2007). Similar models using human subcutaneous fat, a more prominent site of sequestration that closely mimics brain endothelium (Moxon et al., 2013) have yet to be fully realised but are in development (Meehan et al., unpublished). Aside from these limited studies using *P*. *falciparum*, little IVM research has been carried out on parasites using humanised animals, but as this technology develops further, it will likely be utilised by an increasing number of researchers in the future.

## CONFLICTS OF INTEREST

The authors declare no conflicts of interest.

## References

[cmi13023-bib-0001] Akol, G. W. , & Murray, M. (1982). Early events following challenge of cattle with tsetse infected with *Trypanosoma congolense*: Development of the local skin reaction. The Veterinary Record, 110, 295–302. 10.1136/vr.110.13.295 7072104

[cmi13023-bib-0002] Algire, G. H. (1946). An adaptation of the transparent chamber technique to the mouse. Journal of the National Cancer Institute, 4, 1–11. 15393709

[cmi13023-bib-0003] Aliprandini, E. , Tavares, J. , Panatieri, R. H. , Thiberge, S. , Yamamoto, M. M. , Silvie, O. , … Amino, R. (2018). Cytotoxic anti‐circumsporozoite antibodies target malaria sporozoites in the host skin. Nature Microbiology, 3, 1224–1233. 10.1038/s41564-018-0254-z 30349082

[cmi13023-bib-0004] Amino, R. , Giovannini, D. , Thiberge, S. , Gueirard, P. , Boisson, B. , Dubremetz, J. F. , … Ménard, R. (2008). Host cell traversal is important for progression of the malaria parasite through the dermis to the liver. Cell Host & Microbe, 3, 88–96. 10.1016/j.chom.2007.12.007 18312843

[cmi13023-bib-0005] Amino, R. , Thiberge, S. , Martin, B. , Celli, S. , Shorte, S. , Frischknecht, F. , & Ménard, R. (2006). Quantitative imaging of *Plasmodium* transmission from mosquito to mammal. Nature Medicine, 12, 220–224. 10.1038/nm1350 16429144

[cmi13023-bib-0006] Amon, M. , Menger, M. D. , & Vollmar, B. (2003). Heme oxygenase and nitric oxide synthase mediate cooling‐associated protection against TNF‐α‐induced microcirculatory dysfunction and apoptotic cell death. The FASEB Journal, 17, 175–185. 10.1096/fj.02-0368com 12554696

[cmi13023-bib-0007] Arfors, K. E. , Jonsson, J. A. , & McKenzie, F. N. (1970). A titanium rabbit ear chamber: Assembly, insertion and results. Microvascular Research, 2, 516–519. 10.1016/0026-2862(70)90045-2 5523949

[cmi13023-bib-0008] Ariotti, S. , Beltman, J. B. , Chodaczek, G. , Hoekstra, M. E. , van Beek, A. E. , Gomez‐Eerland, R. , … Schumacher, T. N. (2012). Tissue‐resident memory CD8+ T cells continuously patrol skin epithelia to quickly recognize local antigen. Proceedings of the National Academy of Sciences of the United States of America, 109(48), 19739–19744. 10.1073/pnas.1208927109 23150545 PMC3511734

[cmi13023-bib-0009] Beattie, L. , & Kaye, P. M. (2011). Leishmania‐host interactions: What has imaging taught us? Cellular Microbiology, 13, 1659–1667. 10.1111/j.1462-5822.2011.01658.x 21819514

[cmi13023-bib-0010] Belkaid, Y. , Mendez, S. , Lira, R. , Kadambi, N. , Milon, G. , & Sacks, D. (2000). A natural model of Leishmania major infection reveals a prolonged “silent” phase of parasite amplification in the skin before the onset of lesion formation and immunity. Journal of Immunology, 165, 969–977. 10.4049/jimmunol.165.2.969 10878373

[cmi13023-bib-0011] Belkaid, Y. , Valenzuela, J. G. , Kamhawi, S. , Rowton, E. , Sacks, D. L. , & Ribeiro, J. M. (2000). Delayed‐type hypersensitivity to *Phlebotomus papatasi* sand fly bite: An adaptive response induced by the fly? Proceedings of the National Academy of Sciences of the United States of America, 97, 6704–6709. 10.1073/pnas.97.12.6704 10841567 PMC18709

[cmi13023-bib-0012] Benias, P. C. , Wells, R. G. , Sackey‐Aboagye, B. , Klavan, H. , Reidy, J. , Buonocore, D. , … Theise, N. D. (2018). Structure and distribution of an unrecognized interstitium in human tissues. Scientific Reports, 8(1), 4947 10.1038/s41598-018-23062-6 29588511 PMC5869738

[cmi13023-bib-0013] Bouchet, F. , & Lavaud, F. (1999). Solenophagy and telmophagy: Biting mechanisms among various hematophagous insects. Allerg Immunol (Paris), 31, 346–350. 10637663

[cmi13023-bib-0014] Büscher, P. , Bart, J. M. , Boelaert, M. , Bucheton, B. , Cecchi, G. , Chitnis, N. , … Van Reet, N. (2018). Do cryptic reservoirs threaten gambiense‐sleeping sickness elimination? Trends in Parasitology, 34, 197–207. 10.1016/j.pt.2017.11.008 29396200 PMC5840517

[cmi13023-bib-0015] Caljon, G. , Van Reet, N. , De Trez, C. , Vermeersch, M. , Pérez‐Morga, D. , & Van Den bbeele, J. (2016). The dermis as a delivery site of *Trypanosoma brucei* for tsetse flies. PLoS Pathogens, 12, e1005744 10.1371/journal.ppat.1005744 27441553 PMC4956260

[cmi13023-bib-0016] Calvo‐Alvarez, E. , Cren‐Travaillé, C. , Crouzols, A. , & Rotureau, B. (2018). A new chimeric triple reporter fusion protein as a tool for in vitro and in vivo multimodal imaging to monitor the development of African trypanosomes and *Leishmania* parasites. Infection, Genetics and Evolution, 63, 391–403. 10.1016/j.meegid.2018.01.011 29339220

[cmi13023-bib-0017] Capewell, P. , Cren‐Travaillé, C. , Marchesi, F. , Johnston, P. , Clucas, C. , Benson, R. A. , … MacLeod, A. (2016). The skin is a significant but overlooked anatomical reservoir for vector‐borne African trypanosomes. eLife, 5, e17716 10.7554/eLife.17716 27653219 PMC5065312

[cmi13023-bib-0018] Carneiro, M. B. , Hohman, L. S. , Egen, J. G. , & Peters, N. C. (2017). Use of two‐photon microscopy to study *Leishmania major* infection of the skin. Methods, 127, 45–52. 10.1016/j.ymeth.2017.04.012 28434998

[cmi13023-bib-0019] Coombes, J. L. , & Robey, E. A. (2010). Dynamic imaging of host–pathogen interactions in vivo. Nature Reviews. Immunology, 10, 353–364. 10.1038/nri2746 20395980

[cmi13023-bib-0020] Coppi, A. , Natarajan, R. , Pradel, G. , Bennett, B. L. , James, E. R. , Roggero, M. A. , … Sinnis, P. (2011). The malaria circumsporozoite protein has two functional domains, each with distinct roles as sporozoites journey from mosquito to mammalian host. The Journal of Experimental Medicine, 208, 341–356. 10.1084/jem.20101488 21262960 PMC3039851

[cmi13023-bib-0021] De Niz, M. , Ullrich, A.‐K. , Heiber, A. , Blancke Soares, A. , Pick, C. , Lyck, R. , … Spielmann, T. (2016). The machinery underlying malaria parasite virulence is conserved between rodent and human malaria parasites. Nature Communications, 7, 11659 10.1038/ncomms11659 PMC4894950 27225796

[cmi13023-bib-0022] Driskell, R. R. , Lichtenberger, B. M. , Hoste, E. , Kretzschmar, K. , Simons, B. D. , Charalambous, M. , … Watt, F. M. (2013). Distinct fibroblast lineages determine dermal architecture in skin development and repair. Nature, 504, 277–281. 10.1038/nature12783 24336287 PMC3868929

[cmi13023-bib-0023] Dundas, K. , Shears, M. J. , Sun, Y. , Hopp, C. S. , Crosnier, C. , Metcalf, T. , … Wright, G. J. (2018). Alpha‐v–containing integrins are host receptors for the *Plasmodium falciparum* sporozoite surface protein, TRAP. Proceedings of the National Academy of Sciences, 115, 4477–4482. 10.1073/pnas.1719660115 PMC5924908 29632205

[cmi13023-bib-0024] Dutrochet, M. H. (1824). Recherches anatomiques et physiologique sur la structure intime des animaux et des végétaux et sur leur motilité. J. B. Baillière. Paris Paris, 1824, 1–233. PMC5754632 30329773

[cmi13023-bib-0025] Dwinger, R. H. , Rudin, W. , Moloo, S. K. , & Murray, M. (1988). Development of *Trypanosoma congolense*, *T. vivax* and *T. brucei* in the skin reaction induced in goats by infected *Glossina morsitans centralis*: A light and electron microscopical study. Research in Veterinary Science, 44, 154–163. 10.1016/S0034-5288(18)30831-2 3387665

[cmi13023-bib-0027] Erten, A. , Wrasidlo, W. , Scadeng, M. , Esener, S. , Hoffman, R. , Bouvet, M. , & Makale, M. (2010). MR and fluorescence imaging of doxorubicin loaded nanoparticles using a novel in vivo model. Nanomedicine, 6, 797–807. 10.1016/j.nano.2010.06.005 20599526 PMC2980586

[cmi13023-bib-0028] Fishelson, Z. , Amiri, P. , Friend, D. S. , Marikovsky, M. , Petitt, M. , Newport, G. , & McKerrow, J. H. (1992). *Schistosoma mansoni*: cell‐specific expression and secretion of a serine protease during development of cercariae. Experimental Parasitology, 75, 87–98. 10.1016/0014-4894(92)90124-S 1639166

[cmi13023-bib-1090] Flores‐Garcia, Y. , Nasir, G. , Hopp, C. S. , Munoz, C. , Balaban, A. E. , Zavala, F. , Sinnis, P. (2018 Nov 20). MBio, 9(6). pii: e02194–18. 10.1128/mBio.02194-18 PMID: 30459199 PMC6247089 30459199

[cmi13023-bib-0029] Franke‐Fayard, B. , Janse, C. J. , Cunha‐Rodrigues, M. , Ramesar, J. , Büscher, P. , Que, I. , … Waters, A. P. (2005). Murine malaria parasite sequestration: CD36 is the major receptor, but cerebral pathology is unlinked to sequestration. Proceedings of the National Academy of Sciences of the United States of America, 102, 11468–11473. 10.1073/pnas.0503386102 16051702 PMC1183563

[cmi13023-bib-0030] Gaustad, J.‐V. , Brurberg, K. G. , Simonsen, T. G. , Mollatt, C. S. , & Rofstad, E. K. (2008). Tumor vascularity assessed by magnetic resonance imaging and intravital microscopy imaging. Neoplasia, 10, 354–362. 10.1593/neo.08162 18392132 PMC2288537

[cmi13023-bib-0031] Goodwin, L. G. (1970). The pathology of African trypanosomiasis. Transactions of the Royal Society of Tropical Medicine and Hygiene, 64, 797–812. 10.1016/0035-9203(70)90096-9 5495630

[cmi13023-bib-0032] Goodwin, L. G. (1971). Pathological effects of *Trypanosoma brucei* on small blood vessels in rabbit ear‐chambers. Transactions of the Royal Society of Tropical Medicine and Hygiene, 65, 82–88. 10.1016/0035-9203(71)90189-1 5092433

[cmi13023-bib-0033] Gordon, R. M. , & Griffiths, R. B. (1951). Observations on the means by which the cercariae of *Schistosoma mansoni* penetrate mammalian skin, together with an account of certain morphological changes observed in the newly penetrated larvae. Annals of Tropical Medicine and Parasitology, 45, 227–243. 10.1080/00034983.1951.11685493 14915462

[cmi13023-bib-0034] Gueirard, P. , Tavares, J. , Thiberge, S. , Bernex, F. , Ishino, T. , Milon, G. , … Amino, R. (2010). Development of the malaria parasite in the skin of the mammalian host. Proceedings of the National Academy of Sciences of the United States of America, 107, 18640–18645. 10.1073/pnas.1009346107 20921402 PMC2972976

[cmi13023-bib-0035] Haas, W. , Diekhoff, D. , Koch, K. , Schmalfuss, G. , & Loy, C. (1997). Schistosoma mansoni cercariae: Stimulation of acetabular gland secretion is adapted to the chemical composition of mammalian skin. The Journal of Parasitology, 83, 1079–1085. 10.2307/3284366 9406783

[cmi13023-bib-0036] Hellmann, J. K. , Münter, S. , Kudryashev, M. , Schulz, S. , Heiss, K. , Müller, A.‐K. , … Frischknecht, F. (2011). Environmental constraints guide migration of malaria parasites during transmission. PLoS Pathogens, 7, e1002080 10.1371/journal.ppat.1002080 21698220 PMC3116815

[cmi13023-bib-0037] Ho, M. , Hickey, M. J. , Murray, A. G. , Andonegui, G. , & Kubes, P. (2000). Visualization of *Plasmodium falciparum*–endothelium interactions in human microvasculature: Mimicry of leukocyte recruitment. The Journal of Experimental Medicine, 192, 1205–1212. 10.1084/jem.192.8.1205 11034611 PMC2195873

[cmi13023-bib-0038] Hopp, C. S. , Chiou, K. , Ragheb, D. R. T. , Salman, A. M. , Khan, S. M. , Liu, A. J. , & Sinnis, P. (2015). Longitudinal analysis of *Plasmodium* sporozoite motility in the dermis reveals component of blood vessel recognition. eLife, 4, e07789 10.7554/eLife.07789 PMC4594146 26271010

[cmi13023-bib-0039] Ikede, B. O. , & Losos, G. J. (1972). Pathological changes in cattle infected with *Trypanosoma brucei* . Veterinary Pathology, 9, 272–277. 10.1177/030098587200900407 4671499

[cmi13023-bib-0040] Jain, R. , & Weninger, W. (2013). Shedding light on cutaneous innate immune responses: The intravital microscopy approach. Immunology and Cell Biology, 91, 263–270. 10.1038/icb.2012.76 23459295

[cmi13023-bib-0041] Jin, Y. , Kebaier, C. , & Vanderberg, J. (2007). Direct microscopic quantification of dynamics of *Plasmodium berghei* sporozoite transmission from mosquitoes to mice. Infection and Immunity, 75, 5532–5539. 10.1128/IAI.00600-07 17785479 PMC2168273

[cmi13023-bib-0042] Kamhawi, S. , Belkaid, Y. , Modi, G. , Rowton, E. , & Sacks, D. (2000). Protection against cutaneous leishmaniasis resulting from bites of uninfected sand flies. Science, 290(5495), 1351–1354. 10.1126/science.290.5495.1351 11082061

[cmi13023-bib-0043] Kebaier, C. , Voza, T. , & Vanderberg, J. (2009). Kinetics of mosquito‐injected *Plasmodium* sporozoites in mice: Fewer sporozoites are injected into sporozoite‐immunized mice. PLoS Pathogens, 5, e1000399 10.1371/journal.ppat.1000399 19390607 PMC2667259

[cmi13023-bib-0045] Knappe, T. , Mittlmeier, T. , Eipel, C. , Amon, M. , Menger, M. D. , & Vollmar, B. (2005). Effect of systemic hypothermia on local soft tissue trauma‐induced microcirculatory and cellular dysfunction in mice. Critical Care Medicine, 33, 1805–1813. 10.1097/01.CCM.0000172613.74775.C5 16096459

[cmi13023-bib-0046] Lehr, H. a. , Leunig, M. , Menger, M. D. , Nolte, D. , & Messmer, K. (1993). Dorsal skinfold chamber technique for intravital microscopy in nude mice. The American Journal of Pathology, 143, 1055–1062. 7692730 PMC1887078

[cmi13023-bib-0047] Lewert, R. M. , & Lee, C. L. (1954). Studies on the passage of helminth larvae through host tissues: I. Histochemical studies on the extracellular changes caused by penetrating larvae II. Enzymatic activity of larvae in vitro and in vivo. The Journal of Infectious Diseases, 95, 13–51. 10.1093/infdis/95.1.13 13184165

[cmi13023-bib-0049] Matsuoka, H. , Yoshida, S. , Hirai, M. , & Ishii, A. (2002). A rodent malaria, *Plasmodium berghei*, is experimentally transmitted to mice by merely probing of infective mosquito, *Anopheles stephensi* . Parasitology International, 51, 17–23. 10.1016/S1383-5769(01)00095-2 11880224

[cmi13023-bib-0050] McKerrow, J. , & Salter, J. (2002). Invasion of skin by *Schistosoma cercariae* . Trends in Parasitology, 18, 193–195. 10.1016/S1471-4922(02)02309-7 11983589

[cmi13023-bib-0051] Medica, D. L. , & Sinnis, P. (2005). Quantitative dynamics of *Plasmodium yoelii* sporozoite transmission by infected Anopheline mosquitoes. Infection and Immunity, 73, 4363–4369. 10.1128/IAI.73.7.4363-4369.2005 15972531 PMC1168603

[cmi13023-bib-0052] Menger, M. D. , Laschke, M. W. , & Vollmar, B. (2002). Viewing the microcirculation through the window: Some twenty years experience with the hamster dorsal skinfold chamber. European Surgical Research, 34, 83–91. 10.1159/000048893 11867907

[cmi13023-bib-0053] Minkah, N. K. , Schafer, C. , & Kappe, S. H. I. (2018). Humanized mouse models for the study of human malaria parasite biology, pathogenesis, and immunity. Frontiers in Immunology, 9, 807 10.3389/fimmu.2018.00807 29725334 PMC5917005

[cmi13023-bib-0054] Moalli, F. , Ficht, X. , Germann, P. , Vladymyrov, M. , Stolp, B. , de Vries, I. , … Stein, J. V. (2018). The Rho regulator Myosin IXb enables nonlymphoid tissue seeding of protective CD8^+^ T cells. Jem, 215(7), 1869–1890. 10.1084/jem.20170896 PMC6028505 29875261

[cmi13023-bib-0055] Moxon, C. A. , Wassmer, S. C. , Milner, D. A. , Chisala, N. V. , Taylor, T. E. , Seydel, K. B. , … Heyderman, R. S. (2013). Loss of endothelial protein C receptors links coagulation and inflammation to parasite sequestration in cerebral malaria in African children. Blood, 122, 842–851. 10.1182/blood-2013-03-490219 23741007 PMC3731936

[cmi13023-bib-0056] Muthinja, J. M. , Ripp, J. , Krüger, T. , Imle, A. , Haraszti, T. , Fackler, O. T. , … Frischknecht, F. (2018). Tailored environments to study motile cells and pathogens. Cellular Microbiology, 20, e12820 10.1111/cmi.12820 29316156

[cmi13023-bib-0057] Muthinja, M. J. , Ripp, J. , Hellmann, J. K. , Haraszti, T. , Dahan, N. , Lemgruber, L. , … Frischknecht, F. (2017). Microstructured blood vessel surrogates reveal structural tropism of motile malaria parasites. Advanced Healthcare Materials, 6, 1601178 10.1002/adhm.201601178 28117558

[cmi13023-bib-0058] Mwangi, D. M. , Hopkins, J. , & Luckins, A. G. (1990). Cellular phenotypes in *Trypanosoma congolense* infected sheep: The local skin reaction. Parasite Immunology, 12, 647–658. 10.1111/j.1365-3024.1990.tb00994.x 2084609

[cmi13023-bib-0059] Mwangi, D. M. , Hopkins, J. , & Luckins, A. G. (1995). *Trypanosoma congolense* infection in sheep: Ultrastructural changes in the skin prior to development of local skin reactions. Veterinary Parasitology, 60, 45–52. 10.1016/0304-4017(94)00752-X 8644458

[cmi13023-bib-0060] Ng, L. G. , Hsu, A. , Mandell, M. A. , Roediger, B. , Hoeller, C. , Mrass, P. , … Weninger, W. (2008). Migratory dermal dendritic cells act as rapid sensors of protozoan parasites. PLoS Pathogens, 4, e1000222–e1000222. 10.1371/journal.ppat.1000222 19043558 PMC2583051

[cmi13023-bib-0061] Paveley, R. A. , Aynsley, S. A. , Cook, P. C. , Turner, J. D. , & Mountford, A. P. (2009). Fluorescent imaging of antigen released by a skin‐invading helminth reveals differential uptake and activation profiles by antigen presenting cells. PLoS Neglected Tropical Diseases, 3, e528 10.1371/journal.pntd.0000528 19829705 PMC2759291

[cmi13023-bib-0062] Pereira, M. H. , Souza, M. E. L. , Vargas, A. P. , Martins, M. S. , Penido, C. M. , & Diotaiuti, L. (1996). Anticoagulant activity of *Triatoma infestans* and *Panstrongylus megistus* saliva (Hemiptera/Triatominae). Acta Tropica, 61, 255–261. 10.1016/0001-706X(96)00007-1 8790775

[cmi13023-bib-0063] Peters, N. C. , Egen, J. G. , Secundino, N. , Debrabant, A. , Kimblin, N. , Kamhawi, S. , … Sacks, D. (2008). In vivo imaging reveals an essential role for neutrophils in leishmaniasis transmitted by sand flies. Science, 321(5891), 970–974. 10.1126/science.1159194 18703742 PMC2606057

[cmi13023-bib-0065] Pineda, C. M. , Park, S. , Mesa, K. R. , Wolfel, M. , Gonzalez, D. G. , Haberman, A. M. , … Greco, V. (2015). Intravital imaging of hair follicle regeneration in the mouse. Nature Protocols, 10, 1116–1130. 10.1038/nprot.2015.070 26110716 PMC4632978

[cmi13023-bib-0066] Ribeiro, J. M. (1995). Blood‐feeding arthropods: Live syringes or invertebrate pharmacologists? Infectious Agents and Disease, 4, 143–152. 8548192

[cmi13023-bib-0067] Ribeiro, J. M. , Schneider, M. , & Guimaraes, J. A. (1995). Purification and characterization of prolixin S (nitrophorin 2), the salivary anticoagulant of the blood‐sucking bug *Rhodnius prolixus* . The Biochemical Journal, 308, 243–249. 10.1042/bj3080243 7755571 PMC1136869

[cmi13023-bib-0068] Ribeiro, J. M. C. , & Francischetti, I. M. B. (2003). Role of arthropod saliva in blood feeding: Sialome and post‐sialome perspectives. Annual Review of Entomology, 48, 73–88. 10.1146/annurev.ento.48.060402.102812 12194906

[cmi13023-bib-0070] Ritter, U. , Frischknecht, F. , & van Zandbergen, G. (2009). Are neutrophils important host cells for *Leishmania* parasites? Trends in Parasitology, 25, 505–510. 10.1016/j.pt.2009.08.003 19762280

[cmi13023-bib-0071] Rotureau, B. , & Van Den Abbeele, J. (2013). Through the dark continent: African trypanosome development in the tsetse fly. Frontiers in Cellular and Infection Microbiology, 3, 53 10.3389/fcimb.2013.00053 24066283 PMC3776139

[cmi13023-bib-0072] Salter, J. P. , Lim, K. C. , Hansell, E. , Hsieh, I. , & McKerrow, J. H. (2000). Schistosome invasion of human skin and degradation of dermal elastin are mediated by a single serine protease. The Journal of Biological Chemistry, 275, 38667–38673. 10.1074/jbc.M006997200 10993899

[cmi13023-bib-0073] Sandison, J. C. (1924). A new method for the microscopic study of living growing tissues by the introduction of a transparent chamber in the rabbit's ear. The Anatomical Record, 28, 281–287. 10.1002/ar.1090280403

[cmi13023-bib-0074] Sbarbati, A. , Accorsi, D. , Benati, D. , Marchetti, L. , Orsini, G. , Rigotti, G. , Panettiere, P. , 2010 Subcutaneous adipose tissue classification. European Journal of Histochemistry 54, e48 10.4081/ejh.2010.e48 21263747 PMC3167328

[cmi13023-bib-0075] Schuster, J. P. , & Schaub, G. A. (2000). Trypanosoma cruzi: Skin‐penetration kinetics of vector‐derived metacyclic trypomastigotes. International Journal for Parasitology, 30, 1475–1479. 10.1016/S0020-7519(00)00119-3 11428338

[cmi13023-bib-0076] Sidjanski, S. , & Vanderberg, J. P. (1997). Delayed migration of *Plasmodium* sporozoites from the mosquito bite site to the blood. The American Journal of Tropical Medicine and Hygiene, 57, 426–429. 10.4269/ajtmh.1997.57.426 9347958

[cmi13023-bib-0077] Soares, A. C. , Araújo, R. N. , Carvalho‐Tavares, J. , Gontijo, N. d. F. , & Pereira, M. H. (2014). Intravital microscopy and image analysis of *Rhodnius prolixus* (Hemiptera: Reduviidae) hematophagy: The challenge of blood intake from mouse skin. Parasitology International, 63, 229–236. 10.1016/j.parint.2013.07.001 23886517

[cmi13023-bib-0078] Soares, A. C. , Carvalho‐Tavares, J. , Gontijo, N. d. F. , dos Santos, V. C. , Teixeira, M. M. , & Pereira, M. H. (2006). Salivation pattern of *Rhodnius prolixus* (Reduviidae; Triatominae) in mouse skin. Journal of Insect Physiology, 52, 468–472. 10.1016/j.jinsphys.2006.01.003 16580013

[cmi13023-bib-0079] Stewart, M. J. , Nawrot, R. J. , Schulman, S. , & Vanderberg, J. P. (1986). Plasmodium berghei sporozoite invasion is blocked in vitro by sporozoite‐immobilizing antibodies. Infection and Immunity, 51, 859–864. 3512436 10.1128/iai.51.3.859-864.1986 PMC260977

[cmi13023-bib-0080] Stirewalt, M. A. , & Dorsey, C. H. (1974). *Schistosoma manonsi*: Cercarial penetration of host epidermis at the ultrastructural level. Experimental Parasitology, 35, 1–15. 10.1016/0014-4894(74)90002-2 4815016

[cmi13023-bib-0081] Tanowitz, H. B. , Scherer, P. E. , Mota, M. M. , & Figueiredo, L. M. (2017). Adipose tissue—A safe haven for parasites? Trends in Parasitology, 33, 276–284. 10.1016/j.pt.2016.11.008 28007406 PMC5376508

[cmi13023-bib-0082] Teixeira, C. R. , Teixeira, M. J. , Gomes, R. B. B. , Santos, C. S. , Andrade, B. B. , Raffaele‐Netto, I. , … Barral‐Netto, M. (2005). Saliva from *Lutzomyia longipalpis* induces CC chemokine ligand 2/monocyte chemoattractant protein‐1 expression and macrophage recruitment. Journal of Immunology, 175, 8346–8353. 10.4049/jimmunol.175.12.8346 16339576

[cmi13023-bib-0083] Thuita, J. K. , Kagira, J. M. , Mwangangi, D. , Matovu, E. , Turner, C. M. R. , & Masiga, D. (2008). *Trypanosoma brucei rhodesiense* transmitted by a single tsetse fly bite in vervet monkeys as a model of human African trypanosomiasis. PLoS Neglected Tropical Diseases, 2, e238 10.1371/journal.pntd.0000238 18846231 PMC2565695

[cmi13023-bib-0084] Tilg, H. , & Moschen, A. R. (2006). Adipocytokines: Mediators linking adipose tissue, inflammation and immunity. Nature Reviews. Immunology, 6(10), 772–783. 10.1038/nri1937 16998510

[cmi13023-bib-0085] Tong, P. L. , Roediger, B. , Kolesnikoff, N. , Biro, M. , Tay, S. S. , Jain, R. , … Weninger, W. (2015). The skin immune atlas: Three‐dimensional analysis of cutaneous leukocyte subsets by multiphoton microscopy. The Journal of Investigative Dermatology, 135, 84–93. 10.1038/jid.2014.289 25007044 PMC4268113

[cmi13023-bib-0086] Trindade, S. , Rijo‐Ferreira, F. , Carvalho, T. , Pinto‐Neves, D. , Guegan, F. , Aresta‐Branco, F. , … Figueiredo, L. M. (2016). *Trypanosoma brucei* parasites occupy and functionally adapt to the adipose tissue in mice. Cell Host & Microbe, 19, 837–848. 10.1016/j.chom.2016.05.002 27237364 PMC4906371

[cmi13023-bib-0087] van den Abbeele, J. , Caljon, G. , de Ridder, K. , de Baetselier, P. , & Coosemans, M. (2010). Trypanosoma brucei modifies the tsetse salivary composition, altering the fly feeding behavior that favors parasite transmission. PLoS Pathogens, 6, e1000926 10.1371/journal.ppat.1000926 20532213 PMC2880569

[cmi13023-bib-0088] van Zandbergen, G. , Klinger, M. , Mueller, A. , Dannenberg, S. , Gebert, A. , Solbach, W. , & Laskay, T. (2004). Cutting edge: Neutrophil granulocyte serves as a vector for *Leishmania* entry into macrophages. Journal of Immunology, 173, 6521 LP–6525. 10.4049/jimmunol.173.11.6521 15557140

[cmi13023-bib-0089] Vanderberg, J. P. (1974). Studies on the motility of *Plasmodium* sporozoites. The Journal of Protozoology, 21, 527–537. 10.1111/j.1550-7408.1974.tb03693.x 4138523

[cmi13023-bib-0090] Vanderberg, J. P. , & Frevert, U. (2004). Intravital microscopy demonstrating antibody‐mediated immobilisation of *Plasmodium berghei* sporozoites injected into skin by mosquitoes. International Journal for Parasitology, 34, 991–996. 10.1016/j.ijpara.2004.05.005 15313126

[cmi13023-bib-0091] Wagner, R. (1839). Erlauterungstafeln zur Physiologie und Entwicklungsgeschichte (pp. 1–81). Leipzig: Leopold Voss.

[cmi13023-bib-0092] Wang, L. , Bursch, L. S. , Kissenpfennig, A. , Malissen, B. , Jameson, S. C. , & Hogquist, K. A. (2008). Langerin expressing cells promote skin immune responses under defined conditions. Journal of Immunology, 180, 4722 LP–4727. 10.4049/jimmunol.180.7.4722 18354196

[cmi13023-bib-0093] Yamauchi, L. M. , Coppi, A. , Snounou, G. , & Sinnis, P. (2007). Plasmodium sporozoites trickle out of the injection site. Cellular Microbiology, 9, 1215–1222. 10.1111/j.1462-5822.2006.00861.x 17223931 PMC1865575

[cmi13023-bib-0094] Yipp, B. G. , Hickey, M. J. , Andonegui, G. , Murray, A. G. , Looareesuwan, S. , Kubes, P. , & Ho, M. (2007). Differential roles of CD36, ICAM‐1, and P‐selectin in *Plasmodium falciparum* cytoadherence in vivo. Microcirculation, 14, 593–602. 10.1080/10739680701404705 17710630

